# Spontaneous Imbibition and Evaporation in Rocks at the Nanometer Scale

**DOI:** 10.1021/acs.energyfuels.3c02456

**Published:** 2023-11-10

**Authors:** Gijs Wensink, Laurenz Schröer, Helena-Patricia Dell, Veerle Cnudde, Maja Rücker

**Affiliations:** † Department of Mechanical Engineering, Eindhoven University of Technology, Eindhoven 5612 AE, Netherlands; ‡ Department of Geology, Ghent University, Ghent B-9000, Belgium; § Department of Earth Sciences, Utrecht University, Utrecht 3584 CB, Netherlands; ∥ Eindhoven Institute for Renewable Energy Systems, Eindhoven 5612 AZ, Netherlands

## Abstract

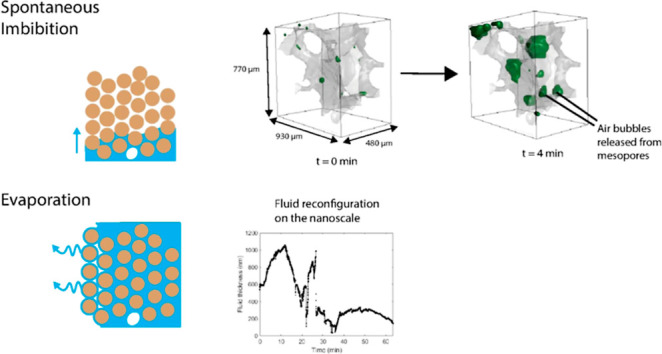

Understanding multiphase fluid displacement dynamics in porous media is of great importance in efficiently designing hydrogen storage projects in porous reservoirs. During gas injection and extraction, cyclic evaporation and spontaneous imbibition processes have an impact on storage efficiency. In both imbibition and evaporation, capillary films on the surface of grains play a role in the transport of water through the pore space. In this study, we use atomic force microscopy to study the formation of these films in carbonate rock during imbibition and their dynamic behavior during evaporation. The imbibition dynamics are related to pore-scale processes determined by micro-CT experiments. We find that imbibition through the mesoporous structure of the grains is slower compared to imbibition in macropores. The formation of the water film on the outer grains is also slower, indicating that a film is evolving due to water flow through intragranular mesopores rather than film flow around the grains. Evaporation experiments reveal that the film shows both local swelling and shrinkage behavior, which we relate to pore-scale processes causing disconnection of the water film. Our results show the close relationship between pore-scale processes and water film dynamics during both spontaneous imbibition and evaporation. This work forms a basis for a more quantitative study of the impact of pore structure on wetting and drying dynamics and can be extended to reactive flow processes.

## Introduction

Storage of natural gas in underground porous reservoirs has been a long-explored means of energy storage. Underground gas storage (UGS) is advantageous compared to storage in overground vessels due to its lower cost and larger volumetric capacity.^1^ In view of energy transition, the focus is shifting toward storage of green hydrogen in reservoirs,^2^ either in pure form or mixed with natural gas.^3^ Natural gas and hydrogen are both immiscible with water, and during injection, they displace the water present in the reservoir. During subsequent extraction, water again replaces the outflowing gas. The multiphase flow processes that take place within the porous rocks during injection and extraction largely determine the efficiency of gas storage.^4^

The displacement dynamics of fluids in porous rocks are controlled by fluid properties, as well as by the structural and chemical properties of the rock,^5,6^ which can vary significantly across length scales.^7^ At the solid–fluid interfaces, molecular interactions between the rock and fluids determine the wetting state of the system. Most natural rocks have an affinity to water,^8^ i.e., forming an interface with water is thermodynamically favorable over forming an interface with gas. This causes water to preferentially reside inside smaller pores and along the pore walls, while gases tend to occupy the centers of large pores.

**Figure 1:**
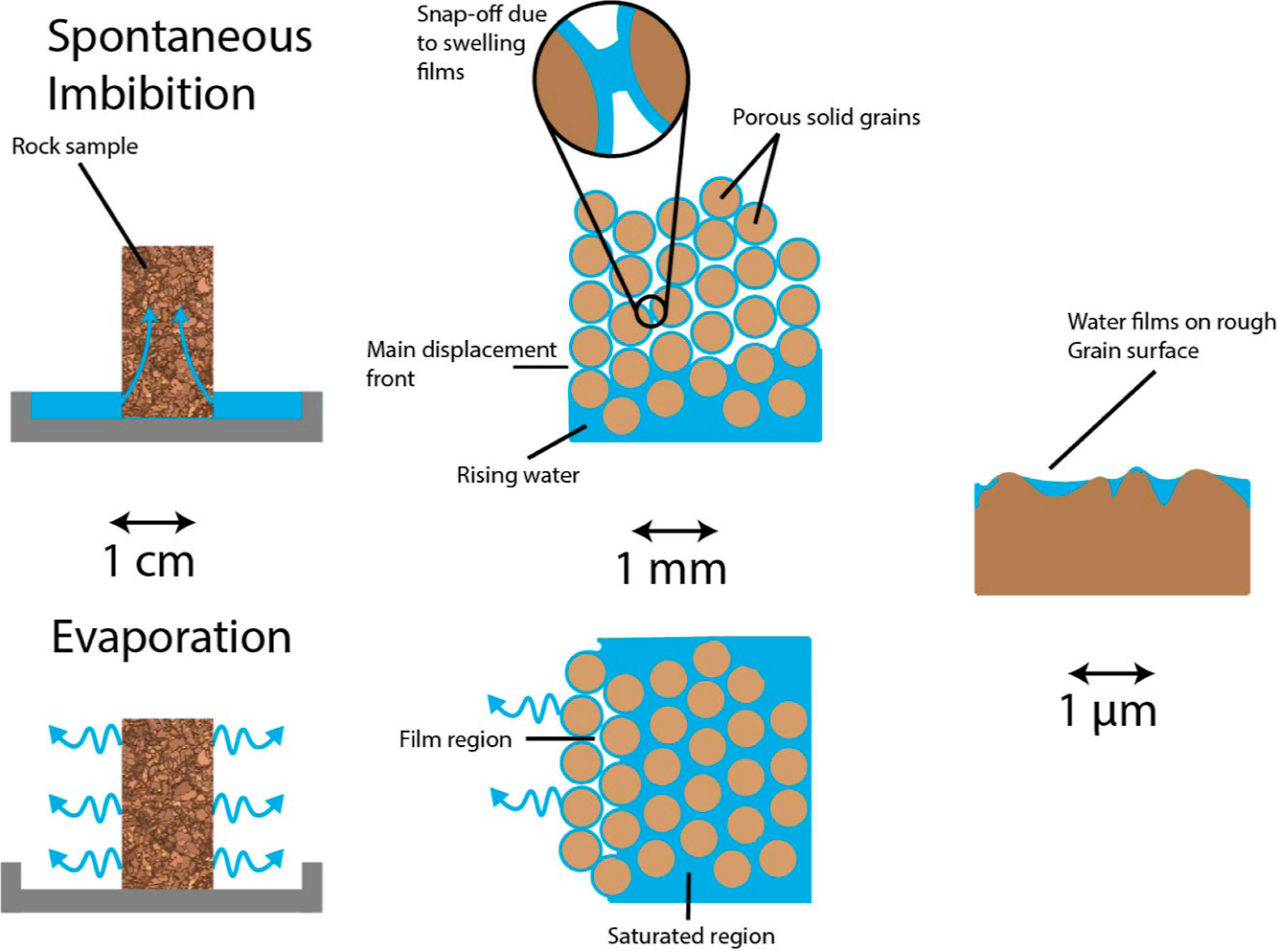
Spontaneous imbibition (top) and evaporation (bottom) in a porous medium. Water films play a role in both processes: in imbibition, swelling of the films ahead of the displacement front may cause snap-off, which could lead to trapping of air inside the pore-space. During evaporation, water films are the main transport medium toward the evaporation front.

Figure 1 schematically shows the fluid distributions inside a simple porous medium, a cylindrical rock sample, during capillary rise (spontaneous imbibition) and during drying (evaporation). After previous wetting of the medium, residual water is often retained in smaller pores and in microscale films along the pore surface.^9^ If the residing gas has low humidity, water may start evaporating, which can induce precipitation of the contained minerals. These precipitates could cause blockage of flow pathways and result in a negative effect on permeability and injectivity of the medium^10−12^ during, e.g., underground storage of dry CO_2_ or hydrogen.

During the capillary rise, water is transported in a main displacement front through the pores. Additionally, residual water films can swell ahead of the main front and cause the formation of capillary bridges inside narrow pore throats when the film flow rate is sufficiently high compared to the movement of the main imbibition front, a process referred to as snap-off^13^ (Figure 1). The snap-off process can cause clusters of gas to remain trapped inside the pores^14,15^ and potentially make a fraction of gas irretrievable.

During evaporation from porous media, three different zones can usually be identified: a dry (gas-saturated) zone, a film zone, and a liquid saturated zone^16^ (Figure 1). The evaporation front, where water is vaporized, forms the boundary between the dry zone and the film zone. Capillary transport through films feeds water from the saturated zone toward the evaporation front.

Understanding the dynamics of the drying and spontaneous imbibition processes is crucial to predicting the behavior of fluids and solids during gas storage projects. Techniques that have been used to monitor fluid distribution during imbibition and evaporation in tight rocks include nuclear magnetic resonance (NMR), microcomputed tomography (micro-CT), and weight-based methods (using balances).^17^ NMR is a useful technique to find fluid distributions in micro-, meso-, and macropores, since they have a distinct relaxation time.^18^ However, NMR is limited in time resolution and in visualizing the displacement processes in 3D. Micro-CT measurements can be employed for 3D visualization^19,20^ but lack the spatial resolution for capturing water film dynamics and fluid distributions in micro- and mesopores, which cannot be disregarded for understanding flow processes in porous media.^21,22^

In this study, we introduce atomic force microscopy (AFM) as a new technique to determine the behavior of water films during drying and imbibition in carbonate rocks. We perform dynamic measurements of the water film thickness across a small area on the outer surface of a Ketton limestone grain during spontaneous imbibition and evaporation. Our goal is to measure the dynamics of nanoscale water films and relate these to pore-scale displacement processes. Micro-CT experiments are used to illustrate the dynamics of spontaneous imbibition at the pore-scale.

## Materials and Methods

### Materials

**Figure 2:**
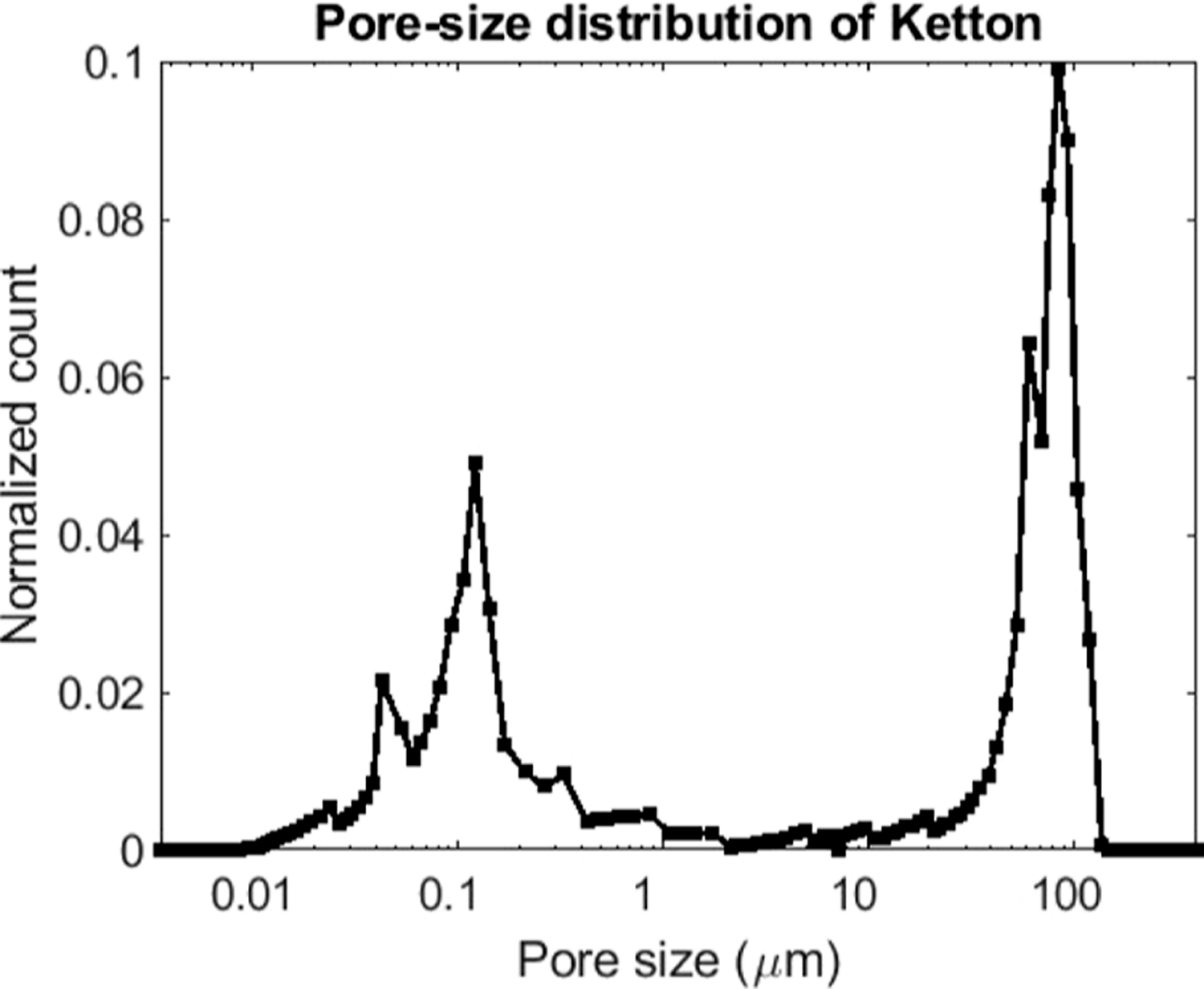
Distribution of pore sizes in Ketton Rock, determined by mercury intrusion porosimetry (MIP).^24^ Reproduced with permission from ref (24), 2018. Copyright CC BY-NC-ND 4.0. Imperial College London.

In our experiment, we made use of cylindrical Ketton rock core samples. Ketton rock is a carbonate with a highly homogeneous chemical composition (99% calcite^23^) and a relatively narrow bimodal pore size distribution,^24^ as shown in Figure 2. The rock contains larger pores (10–200 μm) between mesoporous (10–1000 nm) grains.

The samples used for AFM experiments were disks 1 cm in diameter and 5 mm in height, as required by the sample holder. The samples used for micro-CT experiments were 6 mm in diameter and 2 cm in height. All spontaneous imbibition and evaporation measurements were performed with milli-Q water in a lab environment with a relative humidity between 40 and 60%.

### AFM Imbibition and Evaporation Experiments

**Figure 3:**
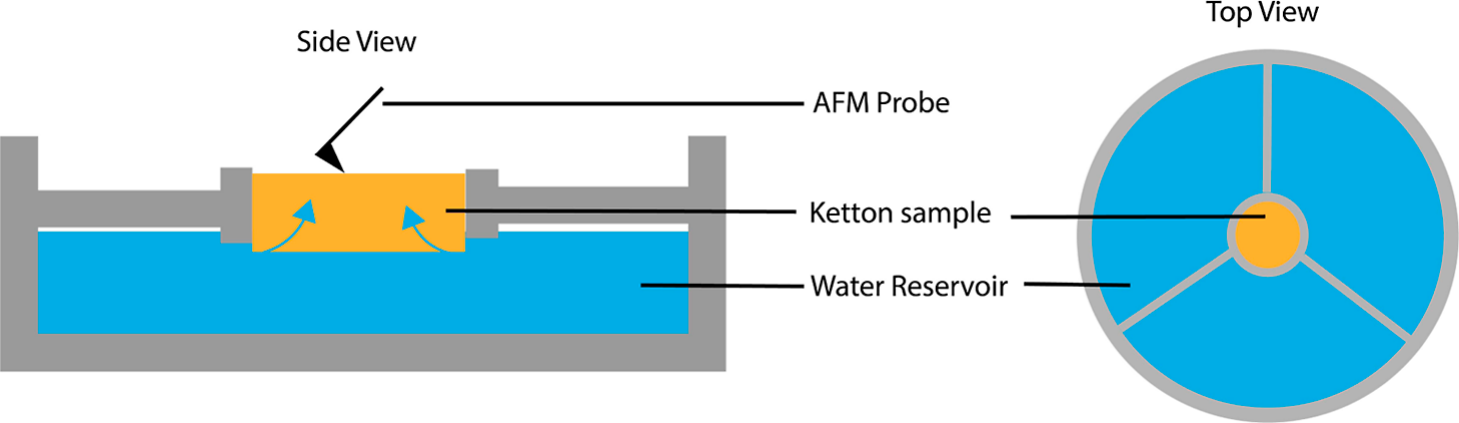
AFM sample holder design. The cylindrical Ketton sample is clamped at the top of the reservoir. The reservoir is filled to the bottom of the sample with water during the imbibition experiment.

To investigate the underlying dynamics of the capillary water film that forms during spontaneous imbibition and disappears during evaporation, we performed AFM measurements. The sample was initiated by placing it in a sample holder with a water reservoir connected to the bottom of the sample. A schematic overview of the sample holder is shown in Figure 3. Through capillary action, water is transported to the top of the sample over time. We measure on the top of a grain at periodic intervals (1, 2, 5, and 14 days) until a water film is detected. To prevent the dry-out of the reservoir, the holder is sealed off from the open air between measurements. After the water film formed, we measured the film thickness during evaporation of the water film by removing the water from the reservoir and scanning in open air while the sample dried out.

**Figure 4:**
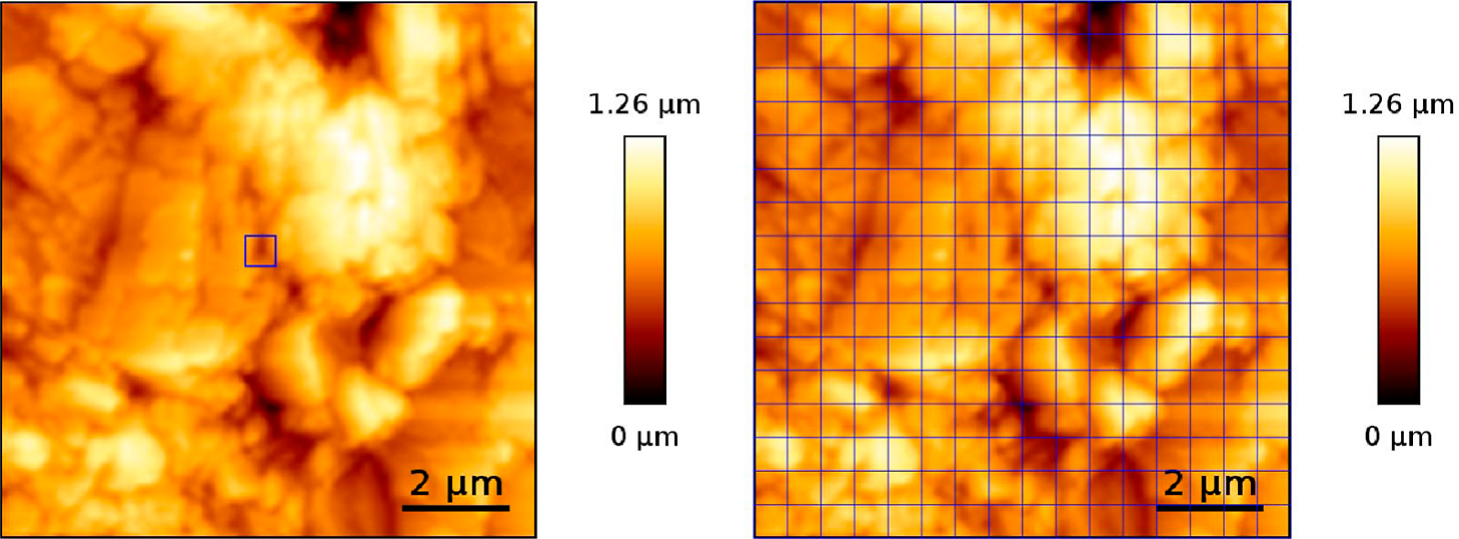
10 × 10 μm example height image of a natural Ketton rock surface scanned by QI-mode imaging. In the right image, we show the grid indicating the points at which a force–distance curve is taken in the force-mapping mode. On the left, the square shows a location where force–distance curves were repeatedly measured in force spectroscopy mode.

We perform our experiments with a JPK instrument bioAFM with a silicon PPP-NCHAuD probe from NANOSENSORS in force mapping mode for imbibition and evaporation experiments and in force spectroscopy mode for an additional evaporation experiment. In these modes, force–distance curves are recorded in a 10 × 10 μm area in a grid (force mapping) or in a single point (force spectroscopy). A tip velocity of 62.5 μm/s was used in the spontaneous imbibition measurements and a velocity of 10 μm/s in the evaporation experiments. A lower velocity was chosen in evaporation experiments to prevent disturbances from tip movement to the water film configuration as much as possible. A 128 × 128 grid of force–distance measurements was used in the spontaneous imbibition measurements for the high spatial resolution needed to capture the formation of a water film. With these settings, one scan could be completed in 5 min. A 16 × 16 grid was used in the evaporation experiments in order to retain a high time resolution. The approach height was adjusted adaptively based on the current film thickness, with a resulting scan time of 3 to 7 min per scan. A sampled height image of the Ketton surface demonstrating where we obtain the force–distance curves is shown in Figure 4. In the figure, we show that, in evaporation experiments, the force–distance curves were mapped continuously in a grid and in a single location.

**Figure 5:**
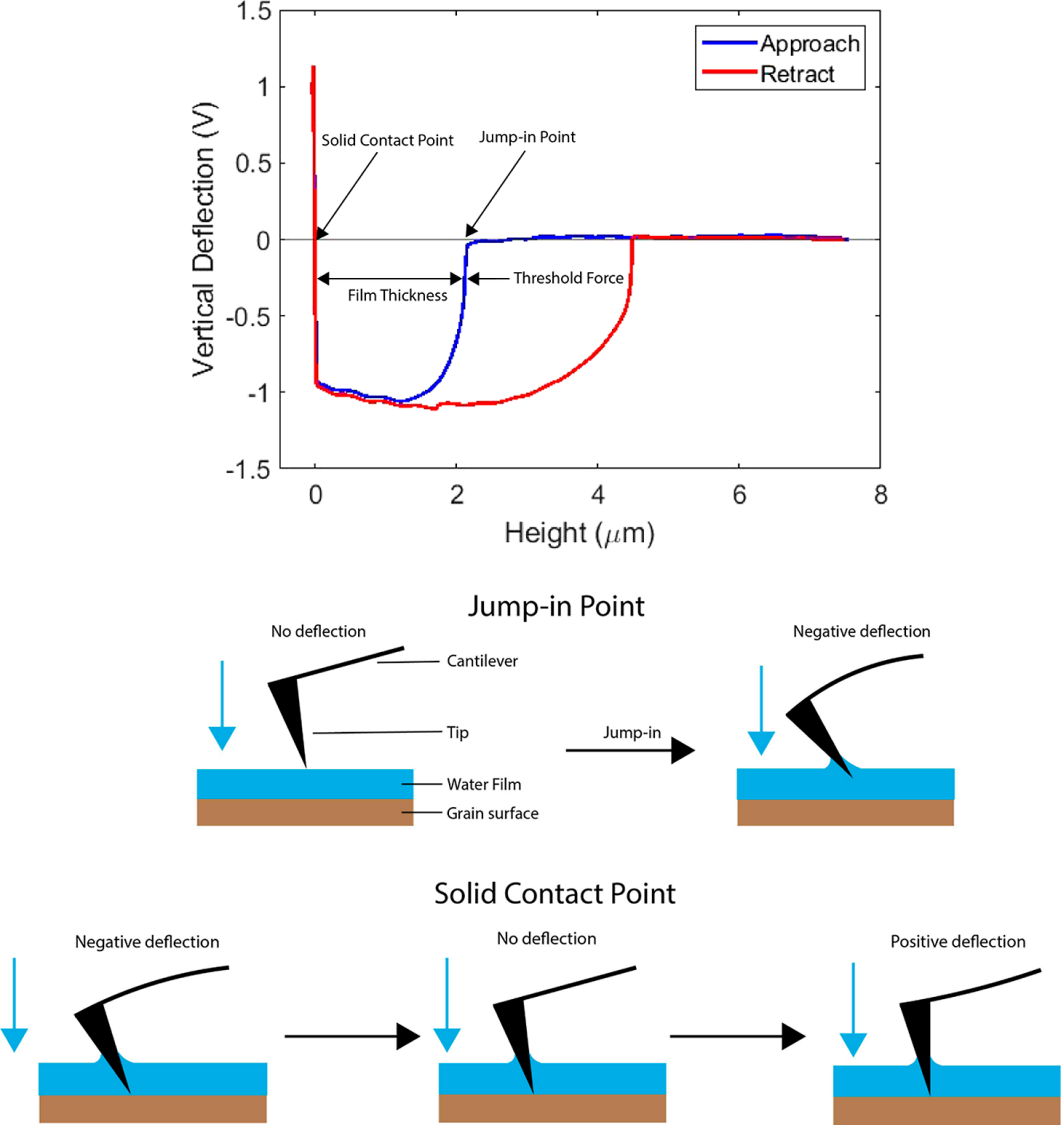
Force–distance curve taken at one of the grid points during dynamic scanning. The force is shown in the form of a vertical deflection signal. As the probe approaches (blue curve), it encounters the water film, which will interact with the probe, causing a jump-in due to capillary action. This is shown as a negative deflection. During further approach, the probe will contact the solid surface, which infers a resisting force, shown as a positive deflection. The difference between probe height at the jump-in point and probe height at the solid contact point is an estimate of the film thickness.^25^ We also show the force–distance curve during retraction (red curve), which shows a negative deflection up to a larger height compared with the approach curve. This hysteresis is due to the capillary neck persisting above the film height.

Before analysis, the recorded force–distance curves are processed in the JPK data processing software by applying baseline leveling to ensure a straight approach curve. In Figure 5, we schematically present the basic concept of how the curves are further processed using an automatic MATLAB (version R2022a) script. The film thickness was determined at each point by identifying a jump-in point, where the tip comes into contact with the water film and capillary forces pull the hydrophilic AFM tip in. Another point is identified, where the tip contacts the solid surface. The difference between these points is the estimated thickness of the film. The same method was previously employed to measure lubricant films,^25^ liquid-like films on ice,^26^ and nanodroplets on a calcite surface.^27^

### Micro-CT Imbibition Experiments

**Figure 6:**
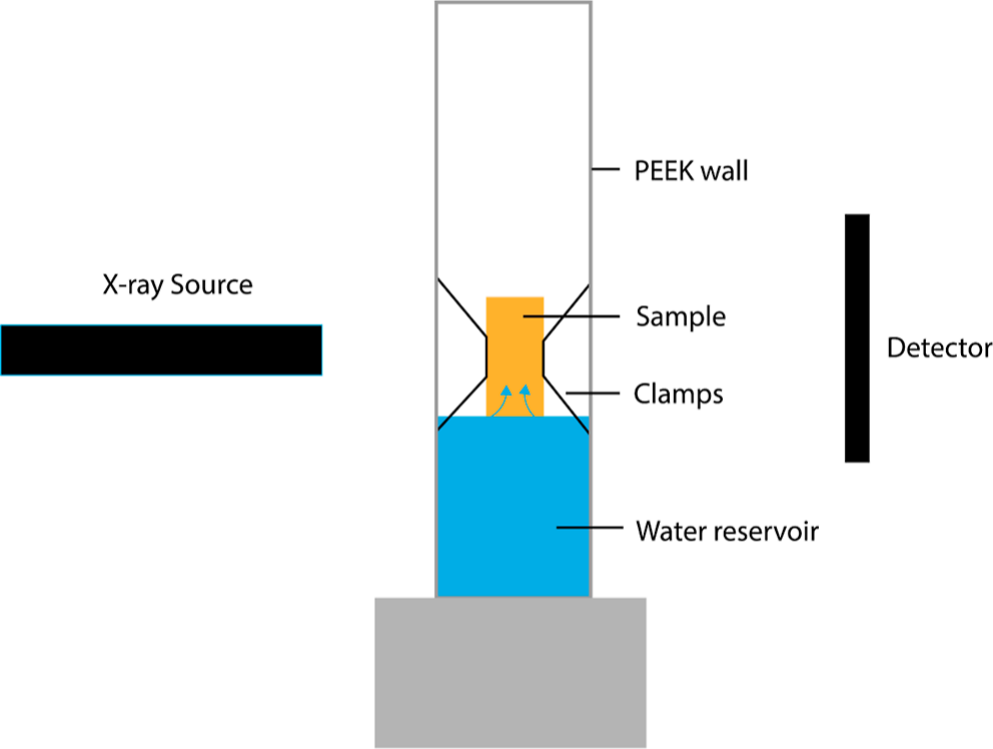
Spontaneous imbibition cell^19^ filled with water up to the bottom of the sample.

Dynamic micro-CT scans of the spontaneous imbibition process were performed on a cylindrical Ketton sample in order to capture the initial fluid exchange processes after primary imbibition. The sample was placed into a cylindrical X-ray transparent PEEK sample holder.^19^ After a dry scan was taken, the cell was filled with ultrapure water to reach the bottom of the sample, as shown in Figure 6.

The micro-CT scans were taken using the EMCT system designed for in situ imaging at the Centre for X-ray tomography at Ghent University (UGCT).^28^ The scans, taken every 2 min to track the dynamics of water clusters inside the sample, were obtained at 110 kV, 8 W, and an exposure time of 70 ms without any filter. 1715 projections were made per scan. The full experiment took 10 min. The scans were reconstructed using Octopus Reconstruction version 8.9.4.9 (XRE). Beam hardening and ring artifact removal were applied. The reconstruction parameters and final gray values were kept constant for each sample to allow comparison of the scans. The reconstruction resulted in 16-bit cross sections (.tiff files) through the sample with a final voxel size of 6.5 μm.

The 3D images were processed further using Dragonfly v2022.1. The image obtained from the dry scan is filtered using a 3-dimensional median filter (spherical, kernel size 5) and the rock grains are identified by selecting a gray value threshold matching the grain region. This image is used as a mask for further scans. Using image registration, the images from the dynamic imbibition scans are rotated and translated to exactly match the mask from the first scan. After masking the grains from the image, the images are filtered with a median filter and segmented using gray-scale thresholding to extract the water and air phases.

## Results

### AFM Experiments

**Figure 7:**
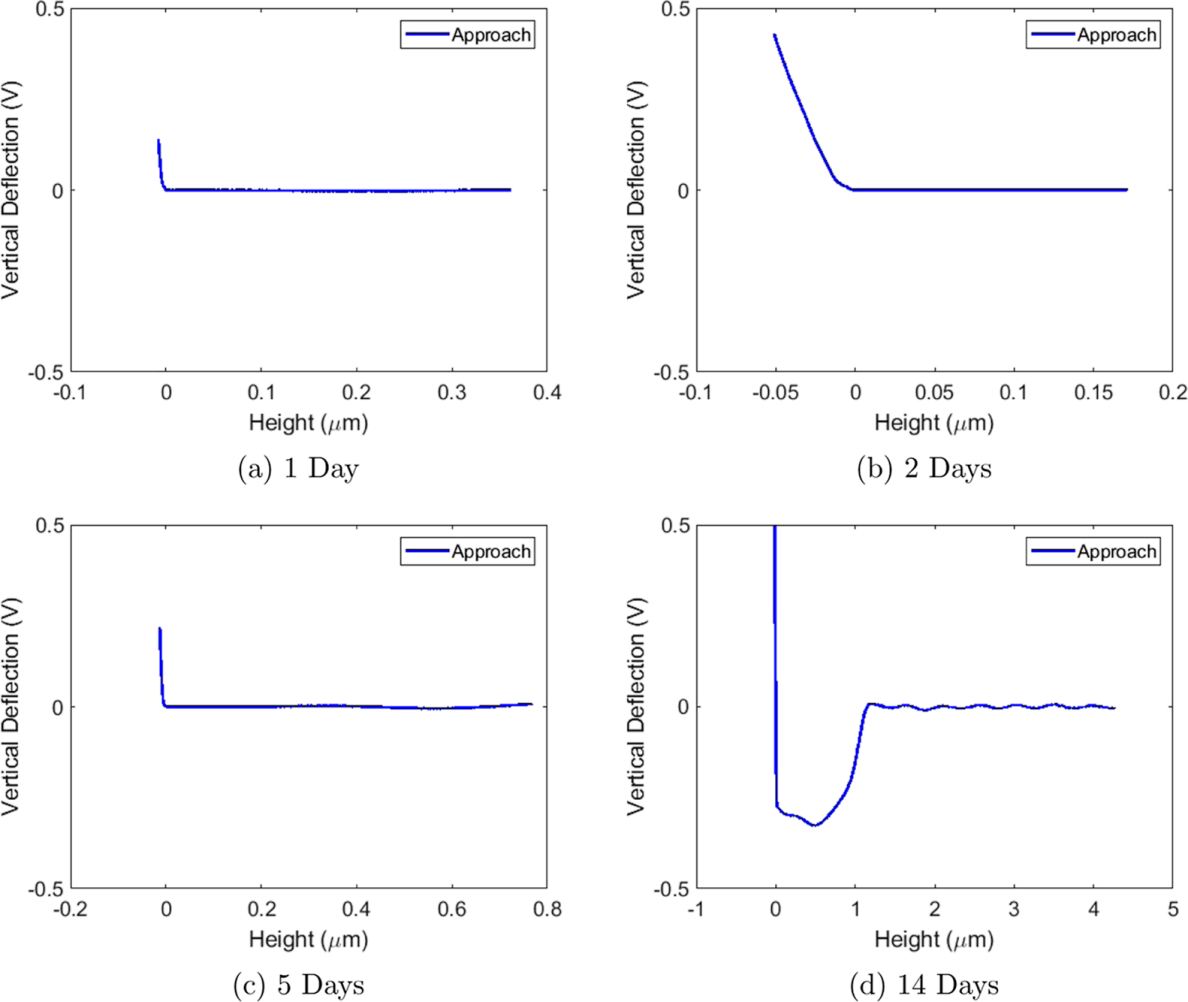
Force–distance curves were captured during spontaneous imbibition at different time intervals. No jump-in point is detected after 1, 2, and 5 days. A scan after 14 days shows a jump-in.

AFM force–distance curve measurements allowed us to distinguish a dry surface from a surface containing a water film. Hence, by periodic measurements we can determine the time required for the water film to form on the outer surface of a Ketton rock grain. Periodic force–distance measurements were performed on a 10 × 10 μm area from the moment we initiated the capillary rise up to the point a film was formed in the middle of an exposed grain. Unexpectedly, we find that in measurements taken after 1, 2, and 5 days, a film could not be observed on the grain surface, as indicated by the sampled force–distance curves shown in Figure 7. A separate measurement after sealing off a sample for 14 days showed a film. Hence, film formation likely takes 6–14 days. The exact period required for the film to form is likely location-dependent, and further study is necessary to investigate this dependence.

**Figure 8:**
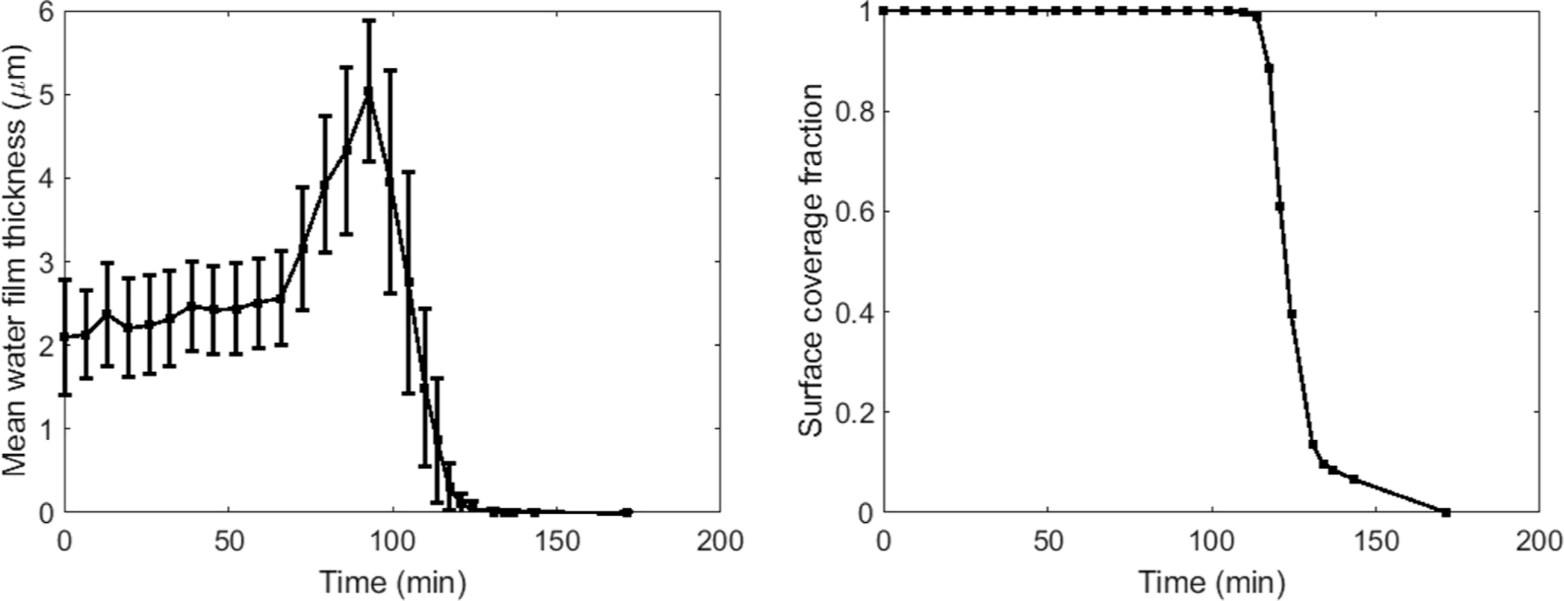
Evolution of mean film thickness (left) and surface coverage fraction (right) during evaporation on a 10 × 10 μm area on the top of a grain of Ketton rock. Error bars in the left figure show the standard deviation of film thickness across the scanned surface.

In Figure 8, we show the evolution of the film thickness and film surface coverage during evaporation of water from the Ketton rock sample over time by force mapping, averaged over a 10 × 10 μm area where we imaged force–distance curves in a 16 × 16 grid. We observe a linear increase in film thickness in the first 65 min, which indicates that water is transported toward the evaporation front at a slightly higher rate than it evaporates. Possibly, the hydrophilic nature of the AFM tip may attract water toward this location. Interestingly, between 65 and 90 min, a sudden larger increase in film thickness is observed, with the film thickness doubling over this period. This increase could be related to water being transported from initially saturated pores when they are displaced by air. After 90 min, the film starts shrinking, and after 100 min, the film coverage starts to decrease. We see that it requires another 50 min for the film to fully disappear. Results of a second evaporation experiment in a different location, which shows different behavior, are shown in the Supporting Information (Figure S2).

**Figure 9:**
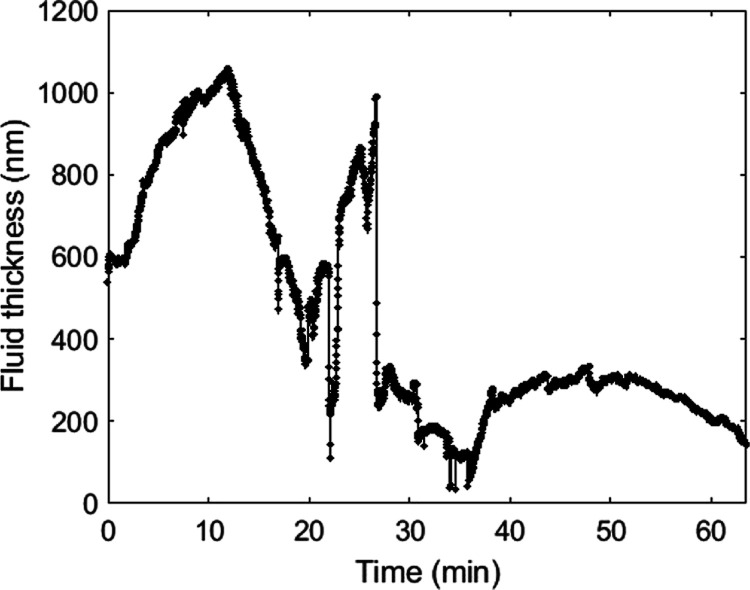
Water film thickness tracked during evaporation at a single point over time.

In Figure 9, we show the evolution of the water film thickness measured at a single point by force spectroscopy. This allows us to capture the film dynamics very locally at a higher time resolution. Compared to the averaged dynamics imaged from the force-mapping experiment, the changes in film thickness are much faster in a single point. Over 1 h of scanning, we observe both sharp (∼10 nm/s) as well as more gradual (∼10 nm/min) increases and decreases in film thickness.

### Micro-CT Imbibition Experiments

**Figure 10:**
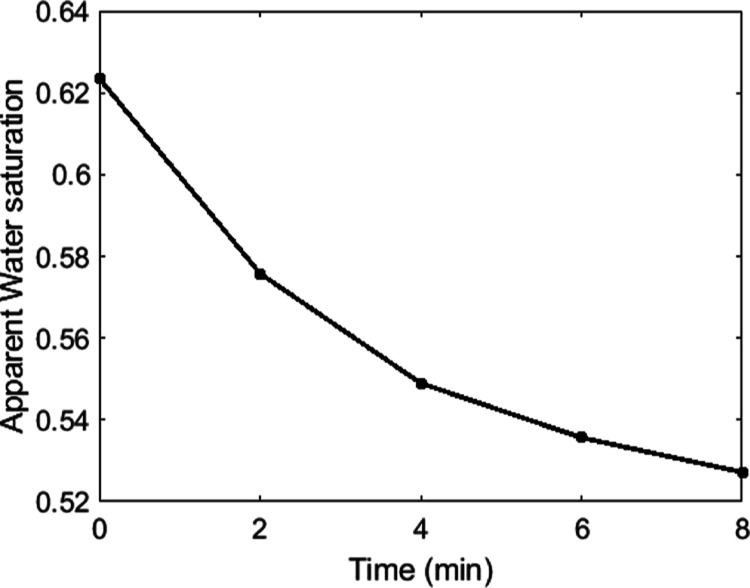
Apparent changes of water saturation in spontaneous imbibition micro-CT experiments calculated from the fluid distribution observed in the macropores.

**Figure 11:**
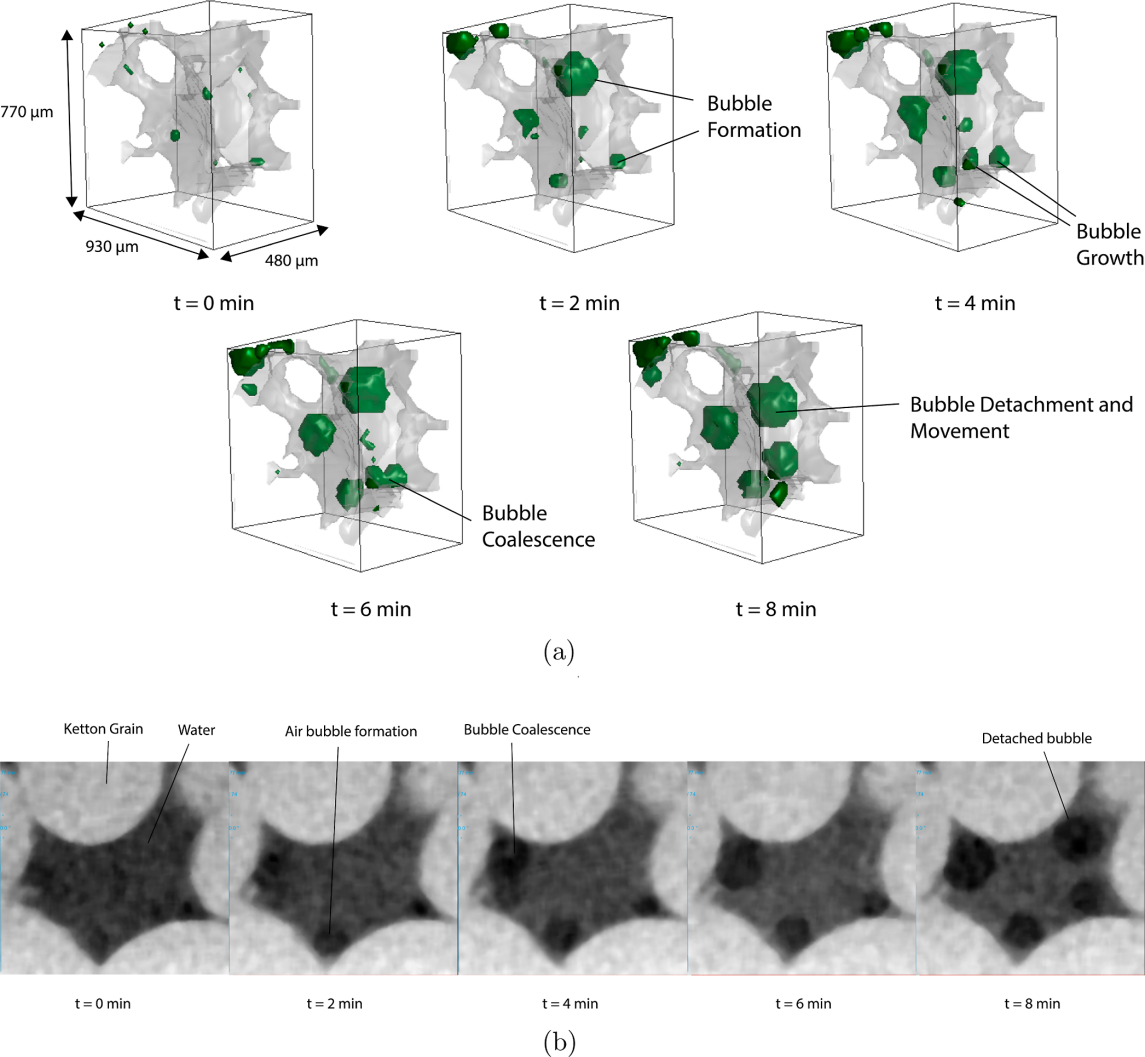
Visualization of a cubic segment extracted from the segmented micro-CT data showing the distribution of air on the inside of a macropore over time.

We conducted dynamic micro-CT to investigate the pore-scale processes that cause the protracted formation of water films on the surface during the AFM scan. In Figure 10, we show the change of the apparent water saturation over time, with the completion of the first scan taken as *t* = 0 min. The macropores of the sample are instantly (within the first scan) filled with 62% water, after which a decreasing apparent water saturation is observed. In Figure 11a, we show 3D images of the air distribution in a pore inside the sample during scanning over time. We observe that initially the pore is filled with water. Over time, we see that air bubbles emerge within the water phase. Since the bubbles are not connected to a continuous gas phase, we expect the air to not originate from connected macropores outside of the field of view. The growing bubbles are attached to the grain surface; hence, we expect that they are formed by air released from the mesopores inside the grains, which are below imaging resolution. Over time, we also observe the coalescence of bubbles and the detachment (Figure 11b) and movement of a larger bubble.

## Discussion

### Dynamics during Spontaneous Imbibition

Our AFM experiments have shown that water films initiate after a period of 2 weeks. As water imbibes into the sample, it will eventually cover the (rough) grains on the outside of the sample with a water film. We believe that this formation of water film is delayed because it is inhibited by the release of air from the mesoporous grains in this location. This is consistent with our observations of emerging air bubbles in the micro-CT experiments.

The capillary rise *h* within a porous medium can be estimated using Jurin’s law^29^
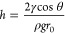
1

with γ the interfacial tension of the water–air surface,^30^ θ the equilibrium contact angle of water on the Ketton surface,^27^ ρ the density of water,^31^*g* the gravitational constant, and *r*_0_ the mean (macro)pore radius (*r*_0_ ≈ *d*/6, with *d* the mean grain diameter). On average, Ketton has a grain diameter of approximately 500 μm. From eq 1, we estimate a capillary rise of 15 cm, which is well above the sample heights. Using the Lucas–Washburn equation,^32^ we can estimate the time *t* needed for the liquid to reach the top of the sample at height *h* (neglecting effects of tortuosity and pore size distribution)
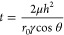
2

with μ the dynamic viscosity of the imbibing liquid.^33^ From eq 2, we estimate that it takes only 0.12 s in order to reach a sample height of 2 cm. This means that the scans of 2 min have insufficient time resolution to capture the imbibition dynamics through the macropores. Hence, with these experiments, we capture the relaxation of the fluids after the initial filling of the macropores by spontaneous imbibition.

Although capillary rise is fast inside the macropores in the inner structure of the Ketton rock, it may take more time for the water to imbibe into the mesoporous grains. Using the Lucas–Washburn equation with a travel distance of 250 μm (estimated radius of a grain), an initial estimate for the time to fill the mesopores in a grain would be only 15 ms. However, the Washburn equation does not take into account the effects of pore-size heterogeneity, tortuosity, and pore connectivity. The imbibition in the mesoporous structure is expected to take place counter-currently for a large part since the surrounding space is completely water-filled. Counter-current imbibition is, in general, slower compared to cocurrent imbibition that takes place in the larger pores, especially considering that trapped gas may have to cross smaller pore throats before being released.^34^

**Figure 12:**
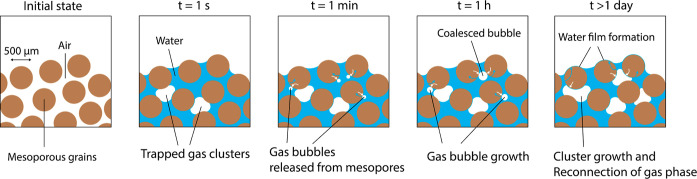
Schematic representation of gas redistribution from mesopores to macropores during spontaneous imbibition at different time scales. Initially, the water imbibes into the larger pores, leaving some gas clusters trapped due to preferential flow paths. Over time, the mesopores are filled, and gas is released back into the larger pores. This causes the gas clusters to grow and reconnect. Transport through the mesoporous grains eventually reached the outer surface, forming a water film along the grain.

The aqueous phase seems to migrate counter-currently from the macropores toward the mesopores. This migration has also been observed in well shut-in experiments in shales using NMR^17^ and has been linked to an increase in permeability of the nonwetting phase because of the potential reconnection of this phase in the larger pores. This is shown schematically in two dimensions in Figure 12. In imbibition experiments using Boise sandstone, which exhibits a bimodal pore distribution similar to Ketton rock,^35^ it was found that supercritical CO_2_ clusters and ganglia would reconnect and grow after water injection was stopped. This may also relate to the release of CO_2_ from the mesoporous grains, although no significant increase in macropore gas saturation was observed. Since our experiments were performed over a shorter time period, we have not observed reconnection of the gas phase, but some coalescence of the released air bubbles is observed that increases the cluster size. We do expect that the porosity fraction of mesopores is sufficiently high to show reconnection of gas flow pathways over an extended time period. To confirm this, dynamic micro-CT scans over a longer time period should be performed in a sequel study.

It is noteworthy to mention that the mechanism of spontaneous imbibition in these bimodally distributed rocks could be impacted by the presence of an initial water saturation. Forced imbibition experiments in Ketton rock^36^ have shown that for partially saturated samples, water preferentially flows through the water-saturated mesopores over the macropores, unlike our observations for initially dry pores.

### Dynamics during Evaporation

The drying of porous media often starts with a constant drying rate period (CRP) followed by a falling rate period (FRP). In the constant drying rate period, evaporation occurs at the outer surface of the porous medium. Water films transport water through the vapor-saturated pores toward this outer surface evaporation front.^16,37,38^ In the FRP, the films have become disconnected from the evaporation front, and the evaporation starts taking place deeper inside the pores. The evaporation rate falls because the process becomes limited by water vapor diffusion from the pores toward the outer surface,^39^ which is typically slower compared to connected film flow. The film dynamics averaged over a 10 × 10 μm area show distinct phases, including a constant film thickness period and a rapidly increasing thickness period. The increasing period could relate to a transition between CRP and FRP, since this is often characterized by the detachment of films from the outer surface,^39^ which could result in a redistribution of water leading to swelling of the remaining films. This redistribution may be partially facilitated by water flow in the mesopores, which have been found to remain saturated during initial stages of drying^12^ and later participate in capillary equilibration, which involves both flow from mesopores to macropores and vice versa.

The standard deviation of film thickness across the 10 × 10 μm area remains relatively constant until the peak thickness is reached. This indicates a rather uniform increase in the film thickness. During the initial drop in thickness, the standard deviation increases, indicating that the film evaporation is less uniform. This observation is another sign that the connectivity of the film decreases and a FRP is initiated since this would leave isolated patches that evaporate at different rates.

In our single-point AFM experiments, we observe both gradual and fast local changes in the film thickness. The gradual changes can be attributed to more gradual filling or emptying of nearby mesopores, while the sharp changes can potentially be associated with fast pore-scale events such as reconnection of the evaporation front with trapped air clusters, as well as contact-point jumps (stick-and-slip motion), which have been observed for droplet evaporation on rough surfaces^40,41^ and may in this case lead to local reconfiguration of the water film.

We note that the AFM measurements are restricted to a 10 × 10 μm area and thus only give a very localized view of the film dynamics. To quantify the relationship between the observed film dynamics and pore-scale events, a more detailed study is needed, where dynamic micro-CT experiments of evaporation along with film dynamic studies on multiple grains within the same sample are used for direct comparison.

## Conclusions

In this study, we present a method for studying the dynamics of nanoscale water films that form on the internal pore surface of Ketton limestone during spontaneous imbibition and evaporation. AFM measurements were successfully applied to track the film thickness and surface coverage over a period of time during the imbibition and consequent evaporation of water from porous Ketton rock. Imbibition experiments show that a water film is initiated after 2 weeks, indicating that the grain surface wetting is slow compared to the time scale of filling of the macropores, typically less than a second. Micro-CT measurements of the spontaneous imbibition process confirm the fast imbibition of the macropores but show a delayed imbibition in intragranular mesopores. Potentially, the formation of the water film on the grain surface is controlled by transport in the mesoporous structure.

During evaporation, the established film initially shows a relatively constant thickness, followed by a period of rapid increase and a dry-out period. These periods can be related to the different drying stages, namely, constant and falling rate periods, which are dictated by film connectivity and pore invasion dynamics. Locally, sudden increases or decreases in thickness are observed that we attribute to surface film reconfiguration as well as events taking place in the mesoporous structure of the grains.

The results from this study show that water films can dynamically respond to displacement events. We expect that the presented technique is suitable to dynamically image the configuration of water films along rough pore surfaces, paving the way for a more quantitative analysis in a follow-up study, for instance, to study cyclic wetting and drying behavior. This can be extended to reactive transport processes, such as mineral dissolution or precipitation from water films and microbial growth in biofilms, which are relevant for future applications of subsurface hydrogen storage in porous reservoirs.

## Supporting Information Available

Fluid and rock properties and results of additional evaporation experiment (PDF)
