# Supplementary material for: Spontaneous Imbibition and Evaporation in Rocks at the Nanometer Scale
Source: Energy Fuels. 2023 Nov 10;37(23):18713–21. doi: 10.1021/acs.energyfuels.3c02456 (PMC10714348; doi:10.1021/acs.energyfuels.3c02456)
Supplement: Supplementary file 1 — ef3c02456_si_001.pdf [file ef3c02456_si_001.pdf]

Supporting Information: Spontaneous Imbibition and Evaporation in Rocks at the Nanometer Scale

Gijs Wensink,[†] Laurenz Schröer,[‡] Helena-Patricia Dell,[†] Veerle Cnudde,[‡,¶] and Maja Rücker[*,†,§]

†*Department of Mechanical Engineering, Eindhoven University of Technology, 5612 AE Eindhoven, Netherlands*

‡*Department of Geology, Ghent University, B-9000 Ghent, Belgium*

¶*Department of Earth Sciences, Utrecht University, 3584 CB Utrecht, Netherlands*

§*Eindhoven Institute for Renewable Energy Systems, 5612 AZ Eindhoven, Netherlands*

E-mail: m.rucker@tue.nl

Rock and Fluid properties

Table S1: Rock and Fluid properties (T = 20°C, p = 1 bar)

Property	Symbol	Value	Unit
Water-air interfacial tension	γ	72.8	mN/m
Water contact angle on Calcite	θ	25	°
Density of water	ρ	998	kg/m^3
Dynamic viscosity of water	μ	1.00	mPa/s

Second AFM evaporation experiment

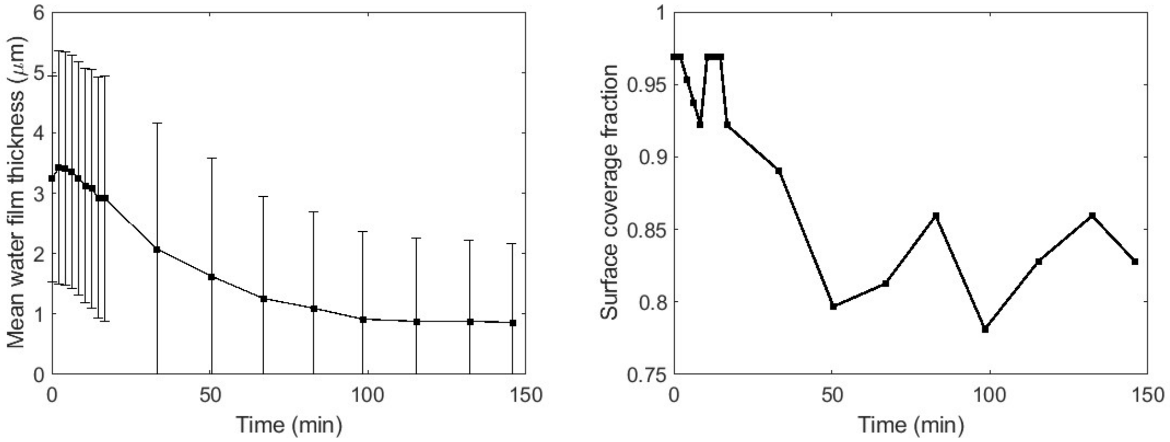

Figure S1: Results of second AFM evaporation experiment. Evolution of mean film thickness (left) and surface coverage fraction (right) during evaporation on a 10x10 μm area on the top of a grain of Ketton rock. Error bars in the left Figure show the standard deviation of film thickness across the scanned surface.

Results of a second AFM experiment are shown in Figure S1. We observe, after a slight increase, only a decrease in film thickness. This indicates that in this location, the film is already getting disconnected at the start of the experiment. The fluctuating surface coverage fraction also could be a result of this. After 150 minutes, the film has not yet disappeared, indicating that the film evaporation takes longer compared to the first experiment. This discrepancy highlights the location dependence of the film evaporation process.
